# Postacute sequelae of COVID-19 at 2 years

**DOI:** 10.1038/s41591-023-02521-2

**Published:** 2023-08-21

**Authors:** Benjamin Bowe, Yan Xie, Ziyad Al-Aly

**Affiliations:** 1 Clinical Epidemiology Center, Research and Development Service, VA Saint Louis Health Care System, Saint Louis, MO USA; 2 Veterans Research and Education Foundation of Saint Louis, Saint Louis, MO USA; 3 Division of Pharmacoepidemiology, Clinical Epidemiology Center, Research and Development Service, VA Saint Louis Health Care System, Saint Louis, MO USA; 4 Department of Medicine, Washington University School of Medicine, Saint Louis, MO USA; 5 Nephrology Section, Medicine Service, VA Saint Louis Health Care System, Saint Louis, MO USA; 6 Institute for Public Health, Washington University in Saint Louis, Saint Louis, MO USA

**Keywords:** SARS-CoV-2, Viral infection, Infectious diseases, SARS virus

## Abstract

Severe acute respiratory syndrome coronavirus 2 (SARS-CoV-2) infection can lead to postacute sequelae in multiple organ systems, but evidence is mostly limited to the first year postinfection. We built a cohort of 138,818 individuals with SARS-CoV-2 infection and 5,985,227 noninfected control group from the US Department of Veterans Affairs and followed them for 2 years to estimate the risks of death and 80 prespecified postacute sequelae of COVID-19 (PASC) according to care setting during the acute phase of infection. The increased risk of death was not significant beyond 6 months after infection among nonhospitalized but remained significantly elevated through the 2 years in hospitalized individuals. Within the 80 prespecified sequelae, 69% and 35% of them became not significant at 2 years after infection among nonhospitalized and hospitalized individuals, respectively. Cumulatively at 2 years, PASC contributed 80.4 (95% confidence interval (CI): 71.6–89.6) and 642.8 (95% CI: 596.9–689.3) disability-adjusted life years (DALYs) per 1,000 persons among nonhospitalized and hospitalized individuals; 25.3% (18.9–31.0%) and 21.3% (18.2–24.5%) of the cumulative 2-year DALYs in nonhospitalized and hospitalized were from the second year. In sum, while risks of many sequelae declined 2 years after infection, the substantial cumulative burden of health loss due to PASC calls for attention to the care needs of people with long-term health effects due to SARS-CoV-2 infection.

## Abstract (web summary)

Analysis of data from the US Department of Veterans Affairs showed that 2 years after severe acute respiratory syndrome coronavirus 2 (SARS-CoV-2) infection, risk for most postacute sequelae remained elevated in people who were hospitalized with COVID-19 but was attenuated in nonhospitalized individuals.

## Main

More than 3 years after the onset of the COVID-19 global pandemic, a wave of evidence suggests that severe acute respiratory syndrome coronavirus 2 (SARS-CoV-2) infection can lead to postacute sequelae in pulmonary and broad array of extrapulmonary organ systems^1–12^—including increased risks and burdens of cardiovascular disorders, neurologic and mental health disorders, metabolic disorders (diabetes and dyslipidemia), kidney disorders and gastrointestinal disorders. The risks and burdens of these sequelae have been assessed in the few months to a year after the onset of infection^1–12^. Few studies with longer follow-ups (longer than 1 year) examined a limited set of symptoms in individuals with COVID-19 or focused exclusively on neurologic sequelae^13–17^. Except for work discussed in ref. ^13^ that mapped risk trajectories of neurologic and psychiatric sequelae and showed substantial heterogeneity in their risk profiles, it remains unclear whether and over what time horizon the risk of postacute sequelae of SARS-CoV-2 attenuates and becomes not significant. A comprehensive assessment of the risks and burdens of postacute sequelae of COVID-19 (PASC) across care settings of the acute infection in the 2 years after the infection is not yet available. Addressing this knowledge gap would deepen our understanding of the postacute and long-term health trajectories of people who had SARS-CoV-2 infection and will inform post-COVID care strategies.

In this study, we use the US Department of Veterans Affairs (VA) national healthcare databases to build a cohort of 138,818 US veterans who survived the first 30 days of SARS-CoV-2 infection and a control group of 5,985,227 users of the US Veterans Health Administration (VHA) with no evidence of SARS-CoV-2 infection. These cohorts were followed longitudinally for 2 years to estimate the risks of death, hospitalization and prespecified array of 80 pulmonary and extrapulmonary sequelae of SARS-CoV-2 throughout the 2-year follow-up and cumulatively at 2 years in mutually exclusive groups according to care setting during the acute phase of the disease (nonhospitalized and hospitalized) and in the overall cohort.

## Results

There were 138,818 and 5,985,227 participants in the COVID-19 and noninfected control groups, respectively. The COVID-19 group had a mean age of 60.91 years (s.d.: 15.96), and 11.41% of participants were female. The control group had a mean age of 62.82 years (s.d.: 16.84), and 9.93% of participants were female. Median follow-up time was 715 days (interquartile range (IQR): 687–720) in the COVID-19 group and 719 days (IQR: 690–720) in the control group, with a total of 255,119 and 11,181,224 person-years of follow-up, respectively, which altogether corresponded to 11,436,344 person-years of follow-up. Demographic and health characteristics of the COVID-19 and noninfected control groups before and after inverse probability weighting for baseline covariates are presented in Supplementary Tables 1 and 2.

We examined risks of death, hospitalization and a set of 80 prespecified postacute COVID-19 sequelae, as well as sequelae aggregated by organ system and aggregated as an overall outcome of PASC by care setting (nonhospitalized (*n* = 118,238) and hospitalized (*n* = 20,580) during the acute phase of SARS-CoV-2 infection) and overall (*n* = 138,818) during the following five time periods of the postacute phase of the disease: 30–90, 91–180, 181–360, 361–540 and 541–720 days after a SARS-CoV-2 infection.

### PASC by acute phase care setting

**Extended Data Fig. 1:**
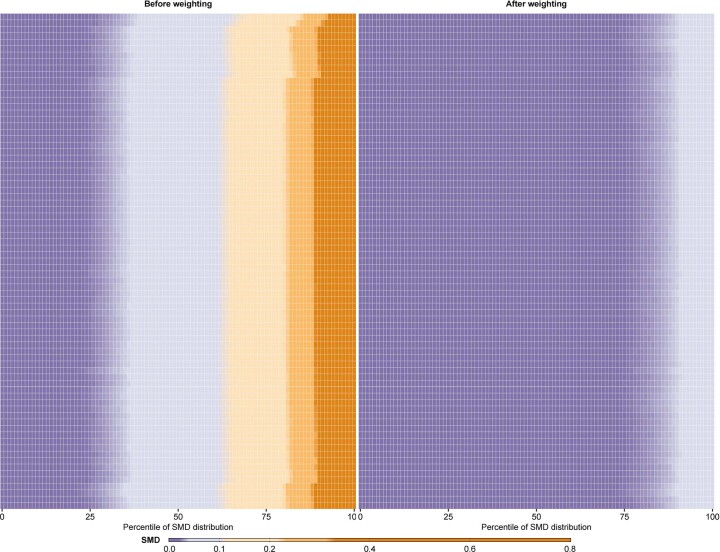
Standardized mean differences between COVID-19 non-hospitalized, hospitalized, and the control before and after weighting in analyses of death, hospitalization, and sequelae. Plots standardized mean differences before weighting (left) and after weighting (right). Each row represents a sub-cohort used in analysis of the risks of death, hospitalization, and sequelae that was free of history of the respective outcome at baseline. Rows are ordered, from top to bottom, on the basis of the lowest to highest percent of SMD that were unbalanced (SMD > 0.1) among unweighted sub-cohorts. Each row consists of the distribution of SMD within the sub cohort. Each cell represents a percentile (from 0th to 100th) of the distribution of the SMD within the respective sub-cohort (x-axis). Includes SMD for non-hospitalized COVID-19 compared to the control, hospitalized COVID-19 compared to the control, and non-hospitalized compared to hospitalized COVID-19. SMD was estimated for differences in participant characteristics including pre-defined covariates, algorithmically selected high dimensional covariates, and non-selected high dimensional covariates. After weighting, no covariates had an SMD greater than 0.1 in any sub-cohorts.

**Extended Data Fig. 2:**
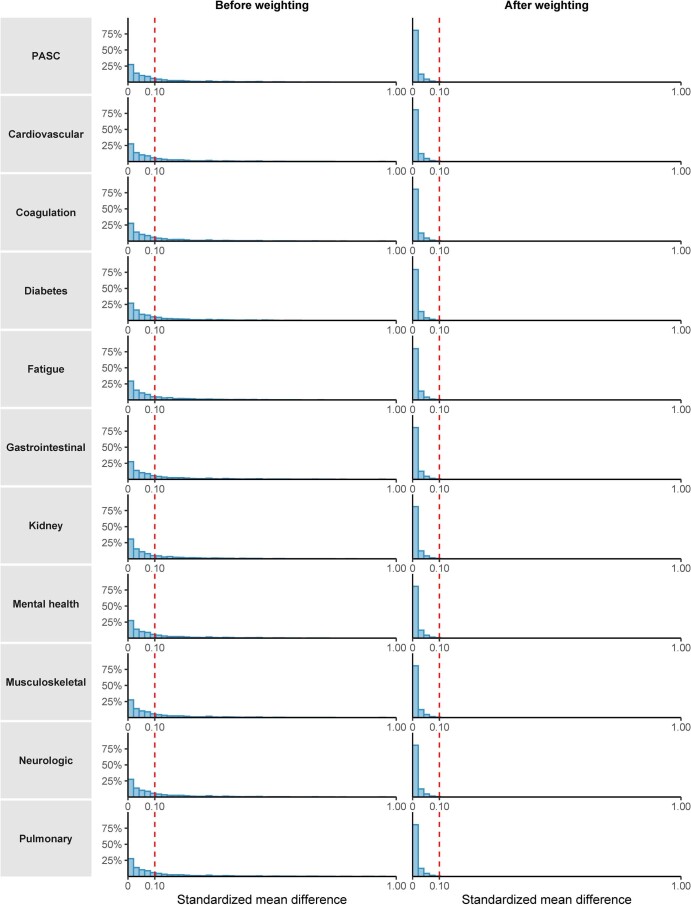
Standardized mean differences between COVID-19 non-hospitalized, hospitalized, and control before and after weighting in analyses of post-acute sequelae (PASC) and sequelae by organ system. Plots includes the standardized mean difference (SMD) (x-axis) and the percent distribution (y-axis), for analyses of post-acute sequelae and sequelae by organ system. Includes differences for non-hospitalized compared to the control, hospitalized compared to the control, and non-hospitalized compared to hospitalized. Covariates examined included pre-defined covariates, algorithmically selected high dimensional covariates, and non-selected high dimensional covariates. An SMD > 0.1 (dotted red line) was taken as evidence of imbalance. After weighting, no SMD were > 0.1.

Participant characteristics by care setting before and after weighting are presented in Supplementary Tables 3 and 4, and assessments of covariate balance are provided in Extended Data Figs. 1 and 2.

#### Risks among nonhospitalized group

**Fig. 1:**
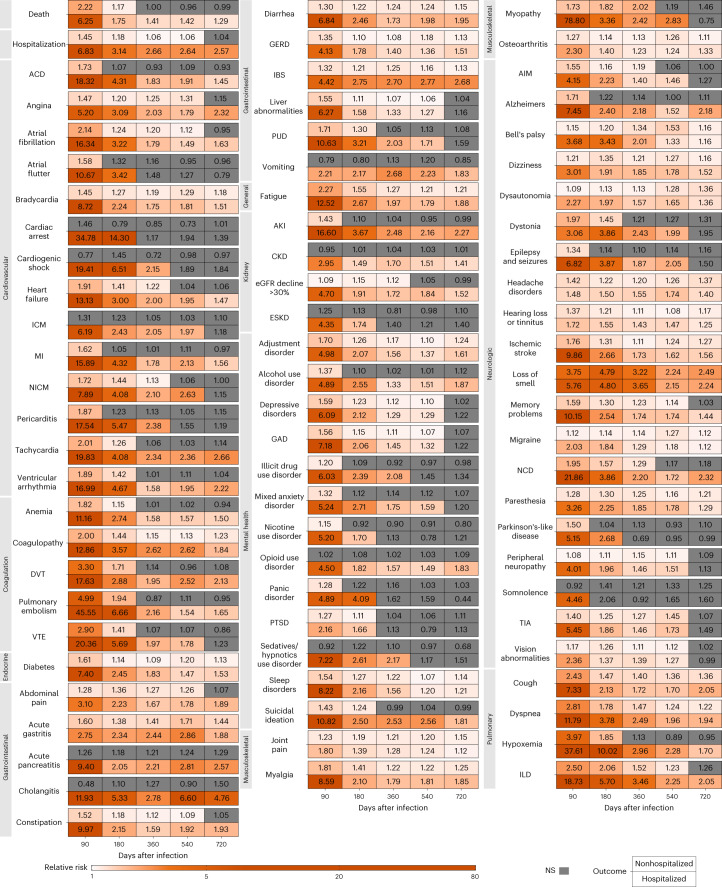
Risk of postacute sequelae of COVID-19 up to 2 years after infection by care setting of the acute phase of the disease. Relative risks by days after infection plotted for time periods of 30–90, 91–180, 181–360, 361–540 and 541–720 days after infection, labeled by the last day of the corresponding time period. Heatmaps include (top row) nonhospitalized for COVID-19 during the acute phase of the disease (*n* = 118,238) corresponding to each sequela and (bottom row) COVID-19 hospitalization during the acute phase of the disease (*n* = 20,580). Relative risks were estimated in comparison to a noninfected control (*n* = 5,985,227). Sequelae are grouped by organ system. ACD, acute coronary disease; AIM, abnormal involuntary movements; AKI, acute kidney injury; CKD, chronic kidney disease; DVT, deep vein thrombosis; ESKD, end-stage kidney disease; GAD, general anxiety disorder; GERD, gastroesophageal reflux disease; IBS, irritable bowel syndrome; ICM, ischemic cardiomyopathy; ILD, interstitial lung disease; MI, myocardial infarction; NCD, neurocognitive decline; NICM, nonischemic cardiomyopathy; PTSD, post-traumatic stress disorder; PUD, peptic ulcer disease; TIA, transient ischemic attack; VTE, venous thromboembolism. NS, non-significant.

**Extended Data Fig. 3:**
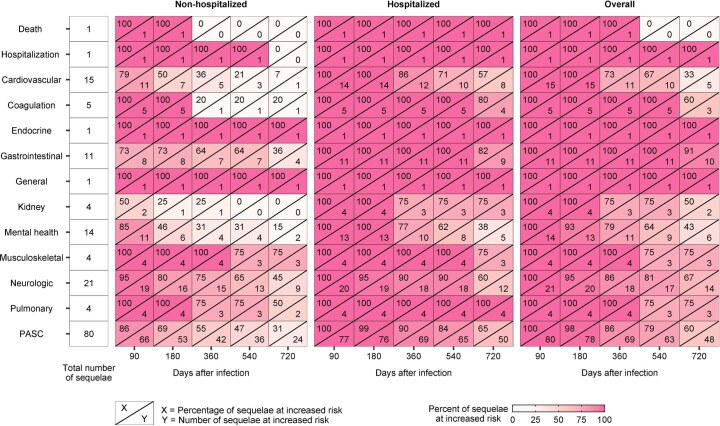
Summary of risk of sequelae by time period of follow-up. Plots present, by time period of analyses, risk summaries of death, hospitalization, sequelae by organ system, and sequelae overall for non-hospitalized COVID-19, hospitalized COVID-19, and overall COVID-19. The column on the left denotes the total number of sequelae examined in the organ system. Within each cell, the top number represent the number of sequelae at increased risk in those with COVID-19 compared to the control, while the bottom number is the percentage at risk among the number of sequelae examined in the organ system.). In analyses by care setting the total number of sequelae examined was 14 cardiovascular sequelae, 13 mental health sequelae, and 20 neurologic sequelae, leading to a total of 77 sequelae examined as part of PASC. PASC, post-acute sequelae of COVID-19.

Risks in the nonhospitalized group are presented in Fig. 1, Extended Data Fig. 3 and Supplementary Table 5. Compared to the control, people with COVID-19 who were not hospitalized during the acute phase of the disease were still at an increased risk of death during the 91–180 days after SARS-CoV-2 infection, but not the 181–720 days following SARS-CoV-2 infection, suggesting the risk horizon occurred between 91 days and 360 days (Fig. 1, Extended Data Fig. 3 and Supplementary Table 5). Compared to those without SARS-CoV-2 infection, the risk of hospitalization was increased in the 361–540 days following the infection, but not the 541–720 days after SARS-CoV-2 infection, suggesting the risk horizon occurred between 361 days and 720 days.

Over the span of 2 years of follow-up in the nonhospitalized group, risks declined and became not significant for 69% of the examined sequelae (Fig. 1, Extended Data Fig. 3 and Supplementary Table 5) and remained increased for 31% (24 of 77) of sequelae—including 7% (1 of 14) of cardiovascular sequelae, 20% (1 of 5) of coagulation and hematologic sequelae, 100% (1 of 1) endocrine sequelae, 36% (4 of 11) of gastrointestinal sequelae, 100% (1 of 1) general sequelae, 0% (0 of 4) of kidney sequelae, 15% (2 of 13) of mental health sequelae, 75% (3 of 4) of musculoskeletal sequelae, 45% (9 of 20) of neurologic sequelae and 50% (2 of 4) of pulmonary sequelae (Extended Data Fig. 3).

**Fig. 2:**
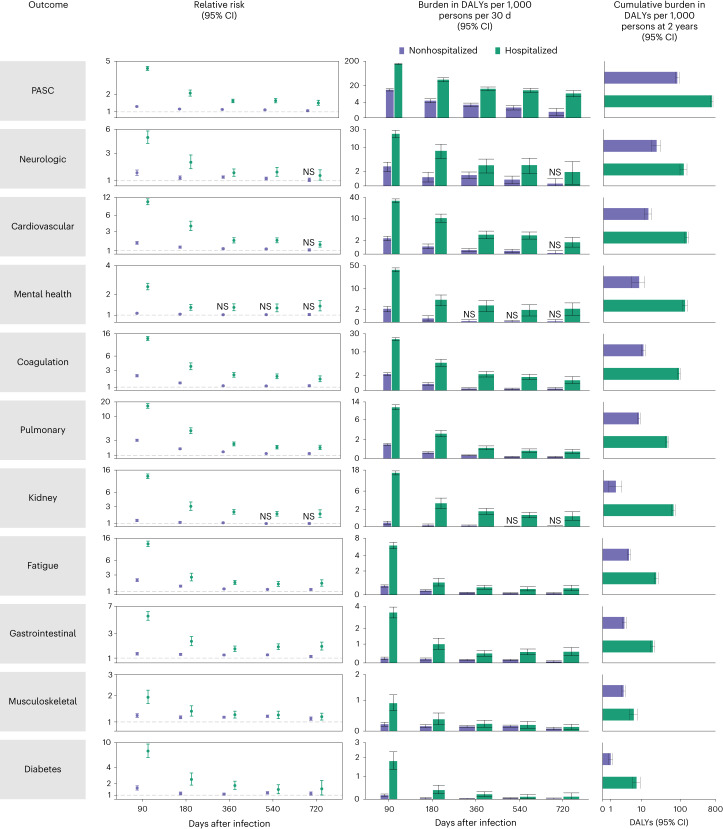
Risks and DALYs of postacute sequelae overall and by organ system by care setting of the acute phase of the disease. The first column includes risk due to COVID-19 of the outcome by time periods of 30–90, 91–180, 181–360, 361–540 and 541–720 days after infection The dot represents the relative risk, while the error bars correspond to the 95% confidence intervals. The second column includes the DALYs rate per 1,000 persons per 30 days by time period. The center of the vertical bar represents the DALYs rate, while the error bars correspond to the 95% confidence intervals. Bar widths differ by the duration of the time period. No adjustment for multiple comparisons was made for the prespecified analyses. Risks and DALYs not significantly different from the control are marked by NS. The third column presents cumulative DALYs per 1,000 persons at 2 years after infection. The center of the horizontal bar represents the cumulative DALYs rate, while the error bars correspond to 95% confidence intervals. Nonhospitalized for COVID-19 (*n* = 118,238), COVID-19 hospitalization (*n* = 20,580) and control group (n = 5,985,227). Outcomes are ordered from top to bottom by largest cumulative DALYs at 2 years after infection in the overall COVID-19 cohort. NS, non-significant.

In analyses of PASC and sequelae by organ system, risks of PASC for coagulation and hematologic disorders, pulmonary disorders, fatigue, gastrointestinal disorders, musculoskeletal disorders and diabetes remained increased 2 years after a SARS-CoV-2 infection in those not hospitalized for COVID-19 compared to those without SARS-CoV-2 infection (Fig. 2 and Supplementary Table 6). The risks of sequelae in several organ systems and disease groups became not significant during the 2 years of follow-up including risks in neurologic (not significant at 541, possible range of risk horizon: 361–720 days), cardiovascular (not significant at 541, 361–720 days), mental health (at 181, 91–360 days) and kidney (not significant at 361, 181–540 days).

**Fig. 3:**
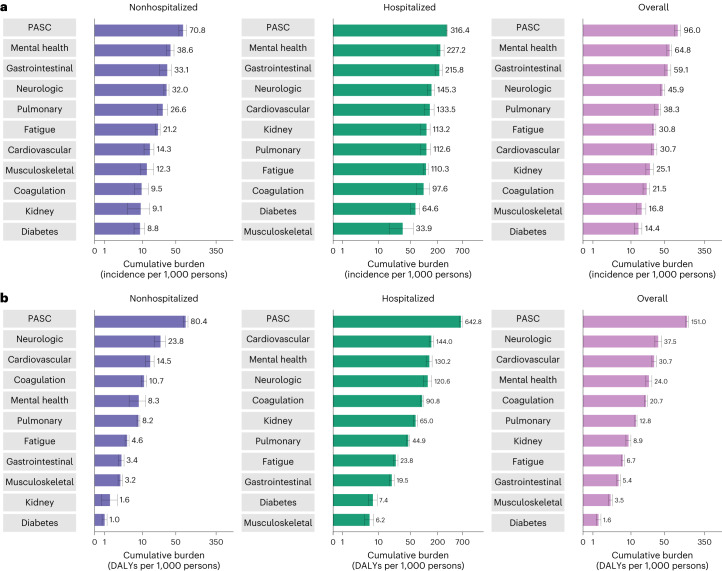
Cumulative incidence and DALYs of postacute sequelae overall and by organ system at 2 years after infection. **a**, Cumulative incidence defined as at least one sequela within that organ system; **b**, cumulative DALYs from sequelae in an organ system. Presented for COVID-19 overall (*n* = 138,818) and by care setting of the acute phase of the disease (nonhospitalized (*n* = 118,238) and hospitalized (*n* = 20,580)) at 2 years after infection. The center of the horizontal bars represents the magnitude of incidence or DALY per 1,000 persons at 2 years after infection and is numerically labeled. Each plot is ordered in a descending fashion. Error bars represent 95% confidence intervals.

**Extended Data Fig. 4:**
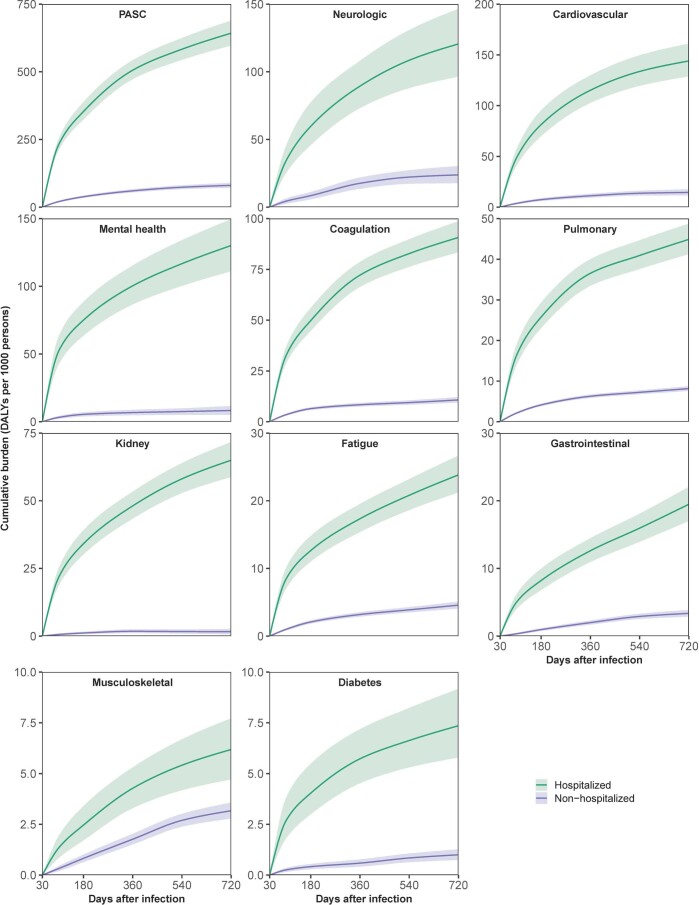
Cumulative disability-adjusted life years (DALYs) of post-acute sequelae and sequelae by organ system in those non-hospitalized and hospitalized with COVID-19. Lines present the cumulative disability adjusted life years (DALYs) due to COVID-19 per 1000 persons (y-axis) by days after infection (x-axis). Bands represent the 95% confidence intervals. Plots are ordered, from left to right and up to down, by cumulative burden at two years after infection. PASC, post-acute sequelae of COVID-19.

Cumulative incident rates of sequelae by organ system in those not hospitalized with COVID-19 are presented in Fig. 3a and Supplementary Table 6, while disability-adjusted life year (DALY) rates are presented in Figs. 2 and 3b, Extended Data Fig. 4 and Supplementary Table 6. There were cumulatively 70.8 (95% confidence interval (CI): 57.8, 84.0) incident PASC per 1,000 persons at 2 years after SARS-CoV-2 infection (Fig. 3a and Supplementary Table 6)—corresponding to 80.4 (95% CI: 71.6, 89.6) DALYs per 1,000 persons at 2 years (Figs. 2 and 3b, Extended Data Fig. 4 and Supplementary Table 6). There were 23.8 (17.7, 30.4) DALYs per 1,000 persons from neurologic disorders, 14.5 (11.6, 17.6) from cardiovascular disorders, 10.7 (9.4, 12.1) from coagulation or hematologic disorders, 8.3 (5.1, 11.6) from mental health disorders, 8.2 (7.6, 8.8) from pulmonary disorders, 4.6 (4.1, 5.1) from fatigue, 3.4 (2.9, 3.9) from gastrointestinal disorders, 3.2 (2.8, 3.6) from musculoskeletal disorders, 1.6 (0.7, 2.7) from kidney disorders and 1.0 (0.7, 1.3) from diabetes (Figs. 2 and 3b, Extended Data Fig. 4 and Supplementary Table 6).

**Fig. 4:**
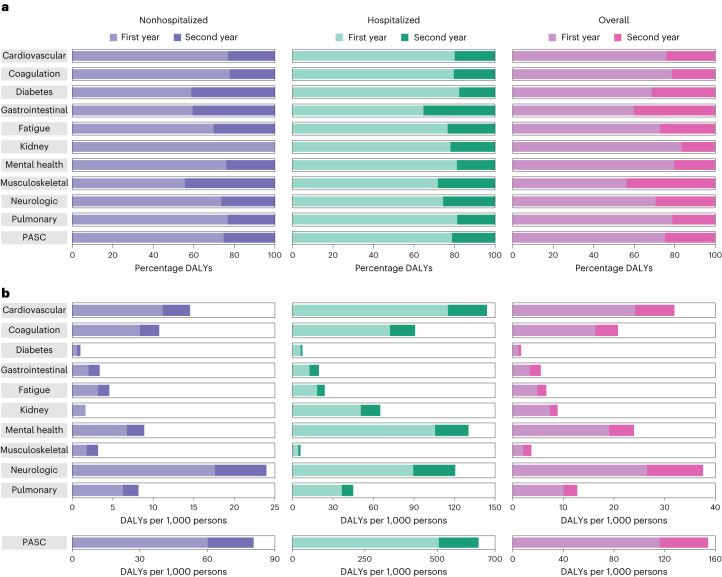
Cumulative DALYs of postacute sequelae overall and by organ system within the first and second years after infection. **a**, Percentage of cumulative DALYs at 2 years contributed from first and second year after infection; **b**, cumulative DALYs per 1,000 persons in the first and second year after infection. Plots presented for nonhospitalized COVID-19 (*n* = 118,238), hospitalized COVID-19 (*n* = 20,580) and overall COVID-19 (*n* = 138,818) compared to control group (*n* = 5,985,227).

Over the span of 2 years of follow-up, 25.3% (18.9, 31.0) of the DALYs due to PASC were contributed from the second year (Fig. 4 and Supplementary Table 6) and 74.7% (69.0, 81.1) contributed from the first year. In total, 23.2% (9.0, 34.1) of the DALYs were due to cardiovascular sequelae, 22.2% (14.6, 28.8) due to coagulation and hematologic sequelae, 41.2% (26.2, 55.3) due to endocrine sequelae, 40.6% (32.7, 48.1) due to gastrointestinal sequelae, 30.2% (23.5, 36.2) due to general sequelae, −9.1% (−112.9, 26.3) due to kidney sequelae, 24.1% (2.4, 39.9) due to mental health sequelae, 44.4% (37.3, 51.3) due to musculoskeletal sequelae, 26.4% (8.9, 40.3) due to neurologic sequelae and 23.2% (19.2, 27.1) due to pulmonary sequelae were contributed from the second year of follow-up (Fig. 4 and Supplementary Table 6).

#### Risks among hospitalized group

Risks in the hospitalized group are presented in Fig. 1, Extended Data Fig. 3 and Supplementary Table 7. Compared to the control, those hospitalized with COVID-19 during the acute phase of the disease remained at increased risk of death and hospitalization through 2 years after SARS-CoV-2 infection (Fig. 1, Extended Data Fig. 3 and Supplementary Table 7); risks were higher in those hospitalized than those not hospitalized (Supplementary Table 8).

Over the 2 years of follow-up, the decline in risk difference between the COVID-19 group and the control group was much less pronounced in people who were hospitalized (than in those nonhospitalized) during the acute phase of SARS-CoV-2 infection (Supplementary Table 8). At 2 years after infection, compared to those without infection, risks declined and became not significant for 35% of sequelae and remained increased for 65% (50 of 77) of sequelae —including 57% (8 of 14) of cardiovascular sequelae, 80% (4 of 5) of coagulation and hematologic sequelae, 100% (1 of 1) endocrine sequelae, 82% (9 of 11) of gastrointestinal sequelae, 100% (1 of 1) general sequelae, 75% (3 of 4) of kidney sequelae, 38% (5 of 13) of mental health sequelae, 75% (3 of 4) of musculoskeletal sequelae, 60% (12 of 20) of neurologic sequelae and 100% (4 of 4) of pulmonary sequelae (Extended Data Fig. 3).

Among those hospitalized with COVID-19, risks remain increased at 2 years after SARS-CoV-2 infection for PASC and all organ systems examined (Fig. 2 and Supplementary Table 9) and were higher in the hospitalized group compared to the nonhospitalized group (Supplementary Table 10). Cumulative incident rates of sequelae by organ system in those hospitalized with COVID-19 are presented in Fig. 3a (Supplementary Table 9), while DALY rates are presented in Figs. 2 and 3b, Extended Data Fig. 4 and Supplementary Table 9. There were cumulatively 316.4 (95% CI: 302.8, 328.2) incident PASC per 1,000 persons at 2 years after SARS-CoV-2 infection (Fig. 3a and Supplementary Table 9)—corresponding to 642.8 (95% CI: 596.9, 689.3) DALYs per 1,000 persons at 2 years (Figs. 2 and 3b, Extended Data Fig. 4 and Supplementary Table 9). These included 144.0 (128.8, 161.1) DALYs per 1,000 persons from cardiovascular disorders, 130.2 (111.2, 149.5) from mental health disorders, 120.6 (96.4, 146.5) from neurological disorders, 90.8 (83.5, 98.7) from coagulation or hematologic disorders, 65.0 (58.8, 71.7) from kidney disorders, 44.9 (41.3, 48.8) from pulmonary disorders, 23.8 (21.2, 26.7) from fatigue, 19.5 (17.0, 22.1) from gastrointestinal disorders, 7.4 (5.8, 9.2) from diabetes and 6.2 (4.7, 7.7) from musculoskeletal disorders (Figs. 2 and 3b, Extended Data Fig. 4 and Supplementary Table 9).

During the follow-up, 21.3% (18.2, 24.5) of the DALYs due to PASC were contributed from the second year (Fig. 4 and Supplementary Table 9) and 78.7% (75.5, 81.8) contributed from the first year. In total, 20.0% (15.8, 24.5) of the DALYs were due to cardiovascular sequelae, 20.4% (16.7, 24.4) due to coagulation and hematologic sequelae, 17.7% (7.0, 29.1) due to endocrine sequelae, 35.4% (29.3, 41.3) due to gastrointestinal sequelae, 23.4% (18.1, 28.9) due to general sequelae, 22.0% (17.4, 26.9) due to kidney sequelae, 18.8% (12.4, 25.5) due to mental health sequelae, 28.2% (14.1, 40.6) due to musculoskeletal sequelae, 25.7% (15.0, 35.9) due to neurologic sequelae and 18.7% (15.7, 22.3) due to pulmonary sequelae were contributed from the second year of follow-up (Fig. 4 and Supplementary Table 9).

### PASC in overall cohort

**Extended Data Fig. 5:**
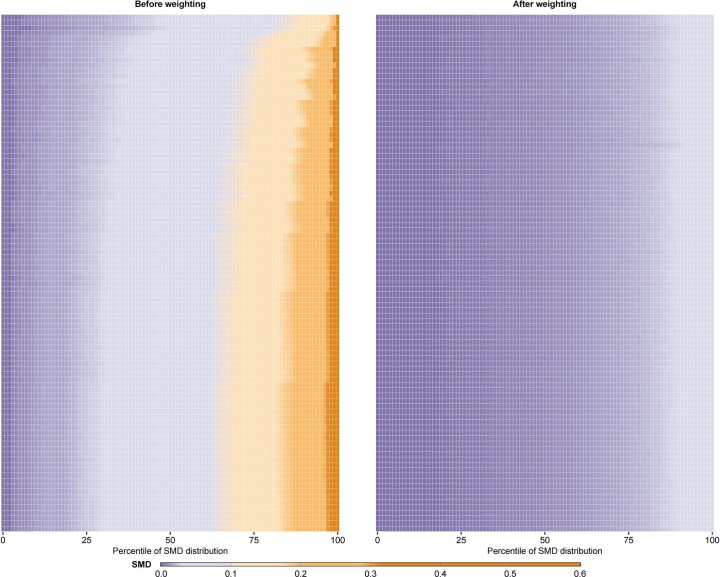
Standardized mean differences between COVID-19 overall and control before and after weighting in analyses of death, hospitalization, and sequelae. Plots standardized mean differences before weighting (left) and after weighting (right). Each row represents a sub-cohort used in analysis of the risks of death, hospitalization, and sequelae that was free of history of the respective outcome at baseline. Rows are ordered, from top to bottom, on the basis of the lowest to highest percent of SMD that were unbalanced (>0.1) among unweighted sub-cohorts. Each row consists of the distribution of SMD within the sub cohort. Each cell represents a percentile (from 0th to 100th) of the distribution of the SMD within the respective sub-cohort (x-axis). Includes SMD for COVID-19 compared to the control. SMD was estimated for differences in participant characteristics including pre-defined covariates, algorithmically selected high dimensional covariates, and non-selected high dimensional covariates. After weighting, no covariates had an SMD greater than 0.1 in any sub-cohorts.

**Extended Data Fig. 6:**
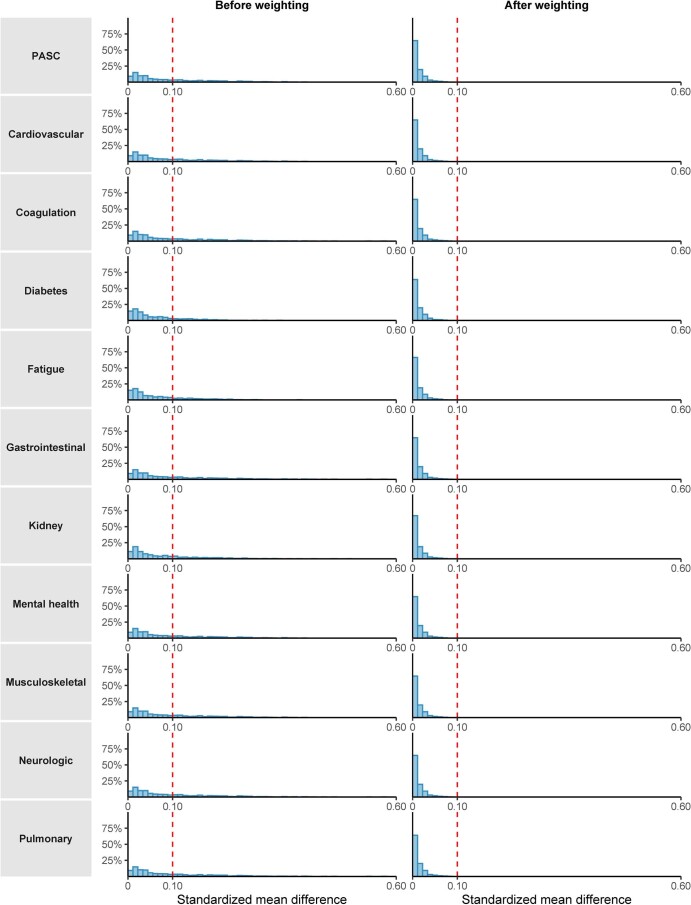
Standardized mean differences between COVID-19 overall and control before and after weighting in analyses of post-acute sequelae (PASC) and sequelae by organ system. Plots includes the standardized mean difference (x-axis) and the percent distribution (y-axis), for analyses of post-acute sequelae and sequelae by organ system. Covariates examined included pre-defined covariates, algorithmically selected high dimensional covariates, and non-selected high dimensional covariates. An SMD > 0.1 (dotted red line) was taken as evidence of imbalance. After weighting, no SMD were > 0.1.

We then examined risks in the overall COVID-19 cohort. Assessment of standardized mean differences (SMDs) after the application of inverse probability weighting suggested that covariates were well balanced between the overall COVID-19 and the control cohort (Extended Data Figs. 5 and 6).

**Fig. 5:**
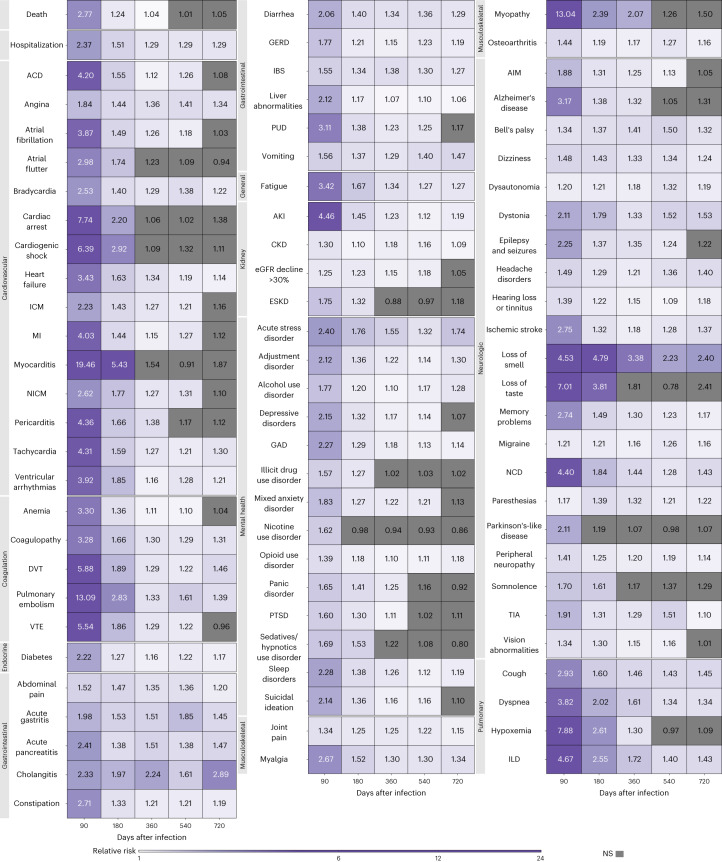
Risk of postacute sequelae of COVID-19 overall up to 2 years after infection. Relative risks by days after infection are plotted for time periods of 30–90, 91–180, 181–360, 361–540 and 541–720 days after infection, labeled by the last day during the corresponding time period. The relative risk is included in the text for each time period and outcome. Relative risks were estimated for overall COVID-19 (*n* = 138,818) in comparison to a noninfected control (*n* = 5,985,227). Sequelae are grouped by organ system. CICM, ischemic cardiomyopathy; KD, chronic kidney disease. NS, non-significant.

Risks of death, hospitalization and incident sequela during each period are provided in Fig. 5, Extended Data Fig. 3 and Supplementary Table 11. Compared to the control group, those with COVID-19 had an increased risk of death in the first 30–360 days after SARS-CoV-2 infection, but not the 361–720 days after SARS-CoV-2 infection, suggesting the risk horizon occurred between 181 days and 540 days (Fig. 5 and Supplementary Table 11). The risk of hospitalization was increased throughout the 2 years after SARS-CoV-2 infection (Fig. 5 and Supplementary Table 11).

We then examined the risks of prespecified incident sequelae, provided in Fig. 5 and Supplementary Table 11. Those who had COVID-19 had a higher risk of all prespecified sequelae in the 30–90 days following SARS-CoV-2 infection. However, as time progressed, the risks of several incident sequelae were attenuated and not statistically significant. Compared to those without COVID-19, those with COVID-19 remained at increased risk of 86% (69 of 80) of sequelae 1 year after SARS-CoV-2 infection. Two years after SARS-CoV-2 infection, those with COVID-19 remained at increased risk of 60% (48 of 80) of the sequelae (Fig. 5, Extended Data Fig. 3 and Supplementary Table 11).

**Fig. 6:**
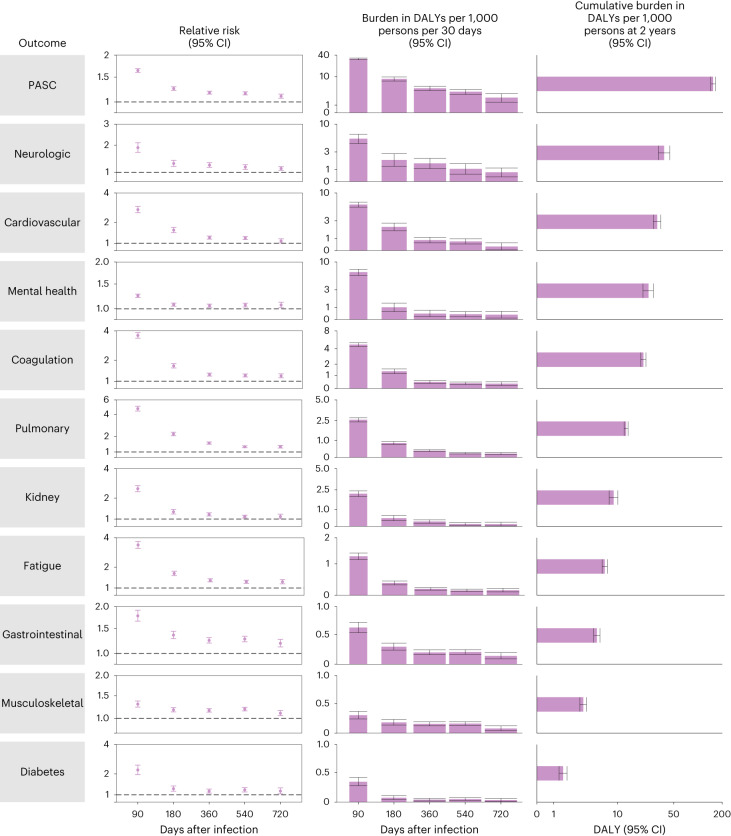
Risk and DALYs of postacute sequelae overall and by organ system in COVID-19 overall. The first column includes risk of the outcome due to COVID-19 by time periods of 30–90, 91–180, 181–360, 361–540 and 541–720 days after infection. The dot represents the relative risk, while the error bars correspond to the 95% confidence intervals. The second column includes the DALYs rate per 1,000 persons per 30 days by time period. The center of the vertical bar represents the DALYs rate, while the error bars correspond to the 95% confidence intervals. Bar widths differ by the duration of the time period. The third column presents the cumulative DALYs per 1,000 persons during the postacute phase at 2 years after infection. The center of the horizontal bar represents the cumulative DALYs rate, while the error bars correspond to 95% confidence intervals. No adjustment for multiple comparisons was made for the prespecified analyses. Outcomes are ordered from top to bottom by the largest cumulative DALYs at 2 years after infection. The horizontal bar represents the cumulative DALYs rate, while the error bars correspond to the 95% confidence intervals. Overall COVID-19 (*n* = 138,818) and control group (*n* = 5,985,227).

Risks of PASC and sequelae by organ system, provided in Fig. 6 and Supplementary Table 12, were highest in the initial postacute phase (30–90 days) and attenuated as follow-up progressed but remained increased in all organ systems 2 years after SARS-CoV-2 infection.

**Extended Data Fig. 7:**
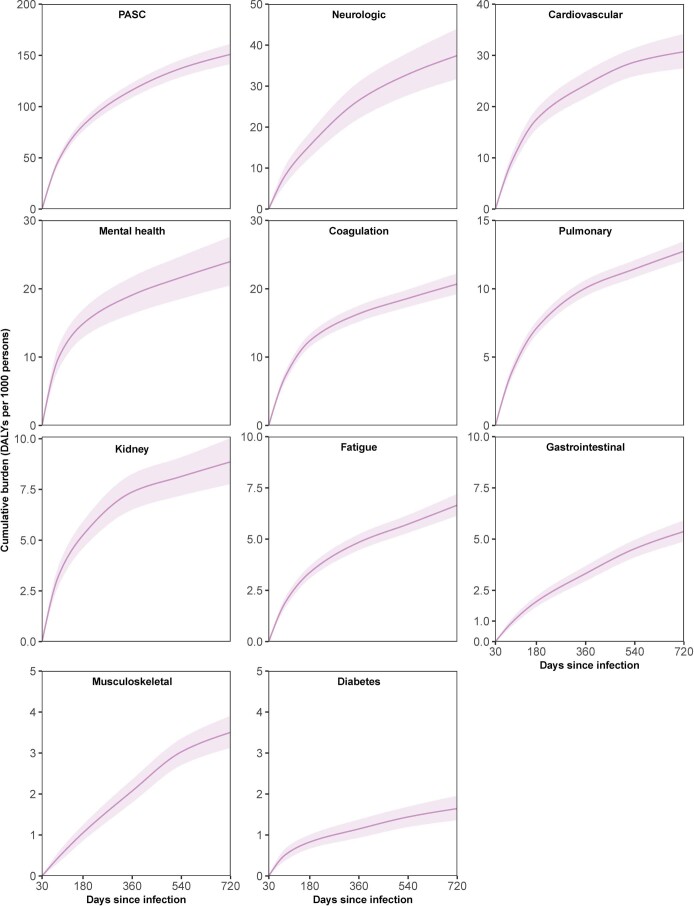
Cumulative disability-adjusted life years (DALYs) of post-acute sequelae and sequelae by organ system in COVID-19 overall. Lines present the cumulative disability adjusted life years (DALYs) due to COVID-19 per 1000 persons (y-axis) by days after infection (x-axis). Bands represent the 95% confidence intervals. Plots are ordered, from left to right and up to down, by cumulative burden at two years after infection. PASC, post-acute sequelae of COVID-19.

There were cumulatively 96.0 (95% CI: 82.5,110.9) incident PASC per 1,000 persons at 2 years after SARS-CoV-2 infection (Fig. 3a and Supplementary Table 12). Cumulative incident rates of sequelae by organ system are presented in Fig. 3a and Supplementary Table 12. DALY rates of sequelae by organ system are presented in Figs. 3b and 5, Extended Data Fig. 7 and Supplementary Table 12. Cumulatively at 2 years after SARS-CoV-2 infection, PASC contributed 151.0 (95% CI: 141.6, 161.0) DALYs per 1,000 persons, including 37.5 (31.8, 44.0) DALYs per 1,000 persons from neurologic disorders, 30.7 (27.5, 34.2) from cardiovascular disorders, 24.0 (20.5, 27.6) from mental health disorders, 20.7 (19.2, 22.3) from coagulation or hematologic disorders, 12.8 (12.1, 13.5) from pulmonary disorders, 8.9 (7.8, 10.0) from kidney disorders, 6.7 (6.2, 7.2) from fatigue, 5.4 (4.9, 5.9) from gastrointestinal disorders, 3.5 (3.1, 3.9) from musculoskeletal disorders and 1.6 (1.4, 2.0) from diabetes (Figs. 3b and Fig. 5, Extended Data Fig. 7 and Supplementary Table 12).

Over the span of 2 years of follow-up, 24.8% (21.6, 27.8) of the DALYs due to PASC were contributed from the second year (Fig. 4 and Supplementary Table 12) and 75.2% (72.2, 78.4) contributed from the first year. In total, 24.1% (18.6, 29.4) of the DALYs were due to cardiovascular sequelae, 21.4% (17.5, 25.2) due to coagulation and hematologic sequelae, 31.4% (21.8, 40.8) due to endocrine sequelae, 40.2% (35.0, 45.3) due to gastrointestinal sequelae, 27.1% (22.7, 31.4) due to general sequelae, 16.7% (9.6, 23.4) due to kidney sequelae, 20.3% (12.9, 27.2) due to mental health sequelae, 43.8% (37.8, 49.7) due to musculoskeletal sequelae, 29.4% (19.5, 38.5) due to neurologic sequelae and 21.2% (18.5, 23.8) due to pulmonary sequelae were contributed from the second year of follow-up (Fig. 4 and Supplementary Table 12).

### Sensitivity analyses

We performed several sensitivity analyses of the risk of postacute sequelae in those with COVID-19 compared to the no-infection control group (Supplementary Table 13). As an alternative to the primary analytic approach that used inverse weighting at baseline to adjust for potential confounding, we used a conditional modeling approach that adjusted for baseline covariates in analyses of each time period to account for potential changes in the composition of the cohort as follow-up progressed (*n* = 6,124,045). In addition to adjusting for baseline covariates through inverse probability weighting, as evidence has suggested that vaccination after infection may be associated with reduced risks of postacute sequelae, we adjusted for vaccination status as a time-varying covariate (*n* = 6,124,045). As an alternative approach to the assessment of the occurrence of outcomes where reinfection contributes additional risks (in addition to those incurred after the primary infection), we additionally censored follow-up of those with COVID-19 at the time of reinfection (*n* = 6,124,045). In addition to the baseline covariates, in consideration of potential differences in healthcare resource utilization between groups, we adjusted for the probability of healthcare resource utilization during follow-up (*n* = 6,124,045). As an additional cohort inclusion criterion, to enhance comparability in receipt of care during follow-up between the COVID-19 group and the control group, we conducted analyses in a subcohort of those that had at least one healthcare encounter per period of analysis during follow-up (*n* = 4,961,017). To examine the influence of iteration of bootstrapping, we conducted 500 iterations of bootstrapping instead of 1,000 iterations used in the primary approach (*n* = 6,124,045). All sensitivity analyses yielded results that were consistent with those produced using the primary approach (Supplementary Table 13).

To further examine the influence of those who were censored during each period on risk estimates, we conducted analyses to estimate the relative risk in those with COVID-19 overall compared to the noninfected control during each time period by including those who were censored in that time period (*n* = 6,124,045). We also used survival models within each time period to estimate the hazard ratios (*n* = 6,124,045). These two additional sensitivity analyses presented in Supplementary Table 14 yielded results consistent with the primary approach presented in Fig. 6.

### Negative outcome control

We examined the association between COVID-19 and incident neoplasms during the 2 years of follow-up as negative outcome control. The analyses suggested no association between COVID-19 infection and the negative outcome control (relative risk of 1.04 (0.95, 1.14), 1.01 (0.92, 1.11), 0.96 (0.90, 1.03) and 1.01 (0.94, 1.10) in the period between 30–90, 91–180, 181–360, 361–540 and 541–720 days, respectively).

## Discussion

In this study involving 138,818 individuals with COVID-19 and 5,985,227 individuals with no known SARS-CoV-2 infection, we estimate the risks of postacute death, hospitalization and a comprehensive array of 80 prespecified sequelae over five prespecified time periods and cumulatively at 2 years. The findings show that among people who were nonhospitalized during the acute phase of SARS-CoV-2 infection (this group represents the majority of people with COVID-19), the risk of death becomes nonstatistically significant at 6 months (possible range of risk horizon: 3–12 months) after infection and the risk of hospitalization remains elevated until 19 months (12–24 months) after infection. The risks of both death and hospitalization remained statistically significantly elevated through the 2 years of follow-up in those who had been hospitalized during the acute phase of SARS-CoV-2 infection. At 2 years, risks remained elevated for 31% and 65% of the sequelae in nonhospitalized and hospitalized individuals, respectively. Cumulatively at 2 years after SARS-CoV-2 infection, PASC contributed 80.4 DALYs per 1,000 persons among nonhospitalized and 642.8 DALYs per 1,000 persons among hospitalized individuals. While most of the DALYs emanated from the first year after infection, a sizable proportion (25.3% in nonhospitalized and 21.3% in hospitalized) was from the second year. Overall, the findings show that while risks of many (but not all) postacute sequelae decline and become nonstatistically significant over time, the decline is less pronounced among those who were hospitalized in the acute phase of infection. The findings highlight the substantial cumulative burden of health loss due to PASC and call for attention to the care needs of people with long-term health effects due to SARS-CoV-2.

Among the nonhospitalized group, risks of 24 sequelae (of 77) remained elevated including several gastrointestinal, musculoskeletal and neurologic sequelae—suggesting a longer-lasting risk horizon for these organ systems. Among hospitalized individuals, the risks of death, hospitalization and 50 sequelae (of 77) representing every organ system remained statistically significantly elevated at 2 years—suggesting the difficult and protracted road to recovery among those whose disease was sufficiently severe to necessitate hospitalization during the acute phase of infection. Taken together the findings suggest that the risk horizon for postacute sequelae after SARS-CoV-2 infection is prolonged even among nonhospitalized individuals and is further prolonged among hospitalized individuals—highlighting the importance of reducing risk of hospitalization among people with SARS-CoV-2 infection (and reinfection) as a means to reduce the risk of long-term health loss^18^.

Measures to reduce the risk of postacute and long-term sequelae in people with SARS-CoV-2 infection should remain the foundation of public health policy. Reducing the risk of infection and transmission with updated vaccines may be a critical strategic avenue to reduce the risk of long-term health loss in populations. Improved uptake of vaccines and antivirals, as well as facilitating access to these across the world, may also help reduce the burden of health loss and stem some of the long-term consequences of SARS-CoV-2 infection. For those who have already been impacted, our results providing a temporal characterization of risks and burdens of 80 sequelae across organ systems may be helpful in informing care pathways (that is, what care people may need and at what time in their illness trajectory) and health system capacity planning^19^. The findings should also be interpreted within the larger body of evidence that has accumulated on the postacute and long-term health effects of SARS-CoV-2. It is clear that the burden of health loss will not only impact patients and their quality of life but also potentially contribute to a decline in life expectancy, and may impact labor participation, economic productivity and societal wellbeing^20–22^.

The findings that SARS-CoV-2 leads to postacute and long-term health effects should be framed within the larger context of infection-associated chronic illnesses—that infections (viral and nonviral) may lead to postacute and chronic disease and that there is likely a bidirectional nexus between noncommunicable diseases and infectious diseases, in that noncommunicable disease often increase the risk of infection and adverse outcomes after infection and that a viral infection may lead to the emergence of new-onset noncommunicable disease^21^.

This study has several strengths. This study comprehensively characterizes the risks of PASC up to 2 years after infection across organ systems and by care setting of the acute phase of the infection. We leveraged the breadth and depth of the vast national healthcare databases of the VA to build a cohort of those with COVID-19 and followed them for 2 years. We used advanced statistical methodologies and adjusted through inverse weighting not only for a set of predefined covariates selected based on prior knowledge but also algorithmically selected high-dimensional covariates from data domains including diagnoses, prescription records and laboratory test results. We designed analyses to examine risks of incident outcomes at multiple different time points throughout the course of 2 years of follow-up and cumulatively. Our estimates of DALYs from sequelae expanded upon prior examinations of the PASC to incorporate not only the number of sequelae but also their influence on health. We performed several sensitivity analyses that varied our modeling approach, cohort inclusion criteria, outcome assessment and adjustment for confounding.

This study has several limitations. The cohort with COVID-19 included those who tested positive for SARS-CoV-2, not including those with a SARS-CoV-2 infection that were not tested, which may have resulted in misclassification of exposure status. Inclusion of these participants in the control group, if their true risk of adverse health outcomes was higher than the noninfected, might result in underestimation of risks. The VA population comprises those that are mostly older and male, which may limit the generalizability of study findings to the nonveteran population. We did not examine the effect within subgroups. While we prespecified 80 sequelae, our analyses did not capture all possible known and yet-to-be-characterized sequelae. We examined all-cause mortality and all-cause hospitalization and did not examine specific causes of mortality or hospitalization. Although we used outcome definitions that rely on multiple data domains including International Classification of Diseases (ICD) codes, medication records and laboratory test results, as well as balanced characteristics of the exposure groups through weighting using a list of predefined and algorithmically selected variables; we cannot completely rule out misclassification bias and residual confounding. Temporal misclassification (that is, misclassification of the timing of occurrence of a sequela) may also be possible. We defined health burden coefficients for DALYs based on the global burden of disease (GBD) study data and methodologies. When a sequela did not directly match a condition in GBD, we applied approximation based on the more aggregated level or detailed level in GBD classification system^23,24^. To obtain sufficient follow-up for the assessment of 2-year outcomes, we enrolled participants until the end of year 2020 (before vaccination became widely available and before alpha, delta or omicron became predominant variants), While we provided summary statistics that rely on statistical significance that depends on statistical power and may vary across study settings, all estimates should be interpreted along with their CIs. Finally, as the characteristics of the pandemic have continued to evolve, as the virus mutates and new variants emerge, as treatment strategies for the acute and postacute phases of the disease are developed and as vaccinations are updated and further deployed, it is possible that the epidemiology of SARS-CoV-2 and its long-term consequences may also evolve dynamically over time.

In sum, our study provides a systematic and comprehensive assessment of the risks of 80 prespecified postacute sequelae. Among nonhospitalized individuals, although the risks of most sequelae became nonstatistically significant at 2 years, substantial risk remains, impacting several major organ systems. The risk horizon for those hospitalized during the acute phase is even longer with persistently increased risk of most sequelae at 2 years. The results may help inform post-COVID care strategies and health system capacity planning to address the postacute and long-term care needs of people with COVID-19.

## Methods

### Ethics statement

This study was approved by the institutional review board of the VA St. Louis Healthcare System, which granted a waiver of informed consent (protocol 1606333).

### Setting

The study was conducted using the electronic healthcare databases of the VA. The VHA, part of the VA, delivers healthcare to discharged Veterans of the US armed forces. The VHA operates the largest national healthcare system in the US, including 146 VA hospitals and 1,269 outpatient sites. Veterans enrolled in the VHA are provided access to a comprehensive package of medical benefits, including preventative and health maintenance, outpatient care, inpatient hospital care, prescriptions, mental healthcare, home healthcare, primary care, specialty care, geriatric and extended care, medical equipment and prosthetics. VHA electronic healthcare databases are updated daily.

### Cohort

**Extended Data Fig. 8:**
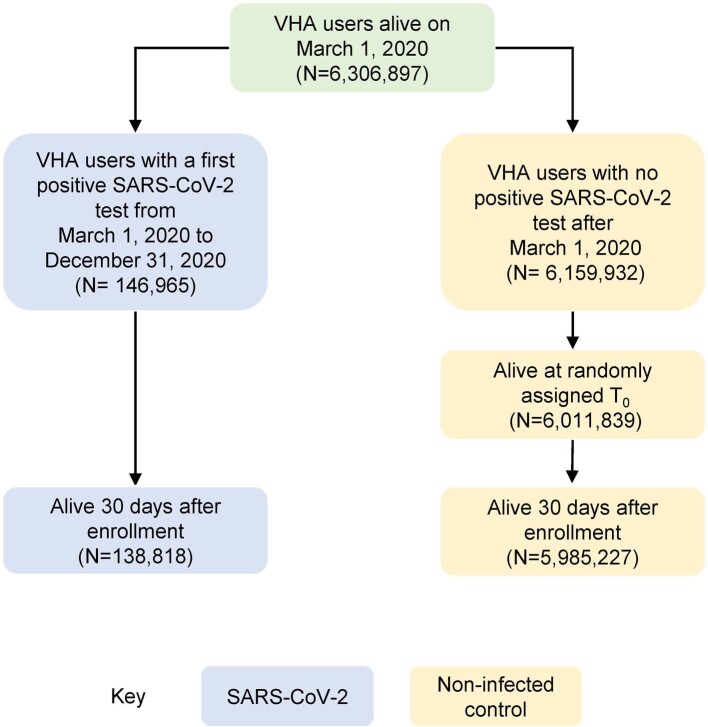
Cohort flowchart. Cohort flowchart of the study.

A flowchart of cohort construction is provided in Extended Data Fig. 8. To construct a group of participants with SARS-CoV-2 infection, we first identified 146,965 users of the VHA with a recorded SARS-CoV-2 infection (defined at the date of first testing positive), between March 1, 2020, and December 31, 2020. Use of the VHA system was defined as having at least two healthcare encounters at the VHA separated by at least half a year in the 2 years before infection. To examine postacute outcomes of COVID-19, we finally selected those alive 30 days after infection resulting in a cohort of 138,818 participants with SARS-CoV-2 infection. The date of infection served as *T*_0_, and follow-up began 30 days after infection.

As a noninfected control, we identified 6,159,932 VHA users between March 1, 2020, and December 31, 2020, with no evidence of a SARS-CoV-2 infection. We then assigned random values for the noninfected controls based on uniform distribution to create randomization. The participants in the control group were assigned a *T*_0_ in the order of the random values on the basis of the distribution of *T*_0_ in those that were SARS-CoV-2 positive. After this assignment, 6,011,839 participants were alive at their assigned *T*_0_. Final control of 5,985,227 consisted of those alive 30 days after their assigned *T*_0_. Among both groups, participants were followed until death, 2 years after *T*_0_, or November 1, 2022.

### Data sources

Data were obtained from the electronic databases of the VHA. Demographic information on age, self-reported race, self-reported sex and death was obtained from the Corporate Data Warehouse (CDW) patient domain. Data on VHA mortality including both deaths inside and outside the hospital are collected from VA and non-VA sources including the VHA’s Beneficiary Identification Record Locator System and medical inpatient datasets, Medicare Vital Status File, Social Security Administration Master File and death certificate information from the National Cemetery Administration^25^. Information on a participant’s geographic location was obtained from the CDW SPatient domain. Information on participant healthcare visits and diagnoses was obtained from the CDW Inpatient and Outpatient domains. Laboratory test results and details were provided by the CDW Laboratory Results domain, while information on medications came from the CDW Outpatient Pharmacy and Bar Code Medication Administration domains. Information on SARS-CoV-2 tests and vaccinations conducted in both VA and non-VA facilities was obtained from the COVID-19 Shared Data Resource. Positive SARS-CoV-2 tests consisted of results from polymerase chain reaction tests or antigen tests conducted in the VA or reported to the VA. Data on inpatient and outpatient Medicare encounters were provided by VA Centers for Medicare and Medicaid Services (VA/CMS). The area deprivation index (ADI) provided a summary measure of contextual disadvantage at a participant’s residential location as a composite of measures of income, education, employment and housing in that location^26^.

### Outcomes

A set of prespecified outcomes were selected and defined on the basis of prior evidence of the PASC^1–12^. A complete list of postacute sequelae examined is provided in Supplementary Table 15. Occurrences of sequelae were identified using definitions that, where applicable, used data from domains including diagnoses codes, laboratory values and medication prescriptions^2–9,27–34^. Risks of each individual sequela were examined in a cohort that had no history of that sequelae in the 2 years before infection (or corresponding *T*_0_). Follow-up occurred starting 30 days after infection and continued until death, 2 years after infection, or November 1, 2022. In analyses by period of time, participants were considered at risk during that period if they had not had the outcome in a prior period. In analysis of kidney outcomes with exception of end-stage kidney disease (ESKD) outcome, participants were censored at the time of ESKD. We also aggregated individual sequelae into composite outcomes based on organ systems, including cardiovascular disorders, coagulation and hematologic disorders, diabetes, fatigue, gastrointestinal disorders, kidney disorders, mental health disorders, musculoskeletal disorders, neurologic disorders and pulmonary disorders, as well as an overall outcome that included all defined PASC. The composite outcome/organ system each sequela was included in is detailed in Supplementary Table 15. Incident occurrence of any sequela within the composite outcome was considered the incident occurrence of the composite outcome. In addition to these sequelae, we also examined death and hospitalization, where hospitalization was allowed to reoccur during each time period of analysis.

### Covariates

To adjust for differences in baseline characteristics between the COVID-19 and control group, we included both a set of predefined covariates and algorithmically selected high-dimensional covariates assessed in the 2 years before *T*_0_ (refs. ^2,3,5–9,35^). Predefined covariates were specified based on prior evidence of variables that may confound associations between COVID-19 and postacute outcomes^2,3^^,^^5–9^^,^^27^^,^^35–39^. Predefined covariates include age, self-reported race (classified into White, Black and Other), self-reported sex, ADI, body mass index (BMI), smoking status (current, former and never) and a series of healthcare utilization parameters. Healthcare utilization parameters included long-term care, receipt of seasonal influenza vaccination, number of inpatient and outpatient Medicare visits in the year before the pandemic, as well as measures of the number of inpatient and outpatient VHA visits, unique medications received from the VHA and number of routine blood chemistry panels recorded at the VHA. Each measure of VHA healthcare utilization was defined as four separate variables, measuring the number of healthcare interactions in the 2 years before infection separated by half-year increments. We also include covariates of anxiety, cardiovascular disease, cerebrovascular disease, chronic kidney disease, chronic lung disease, dementia, depression, diabetes, immunocompromised status and peripheral artery disease. Immunocompromised status was defined (according to the CDC definition) by a history of organ transplantation, advanced kidney disease (an estimated glomerular filtration rate (eGFR) less than 15 ml min^−1^ 1.73 m^−^^2^ or end-stage renal disease), cancer, human immunodeficiency virus or conditions with prescriptions of more than 30 days use of corticosteroids or immunosuppressants including medications used to treat systemic lupus erythematosus and rheumatoid arthritis. We adjusted for eGFR, systolic and diastolic blood pressure prior and closest to *T*_0_. eGFR was calculated using the new Chronic Kidney Disease-Epidemiology Collaboration creatinine equation based on serum creatinine, age and sex^40^. To account for spatiotemporal differences, we adjusted for the week of infection or corresponding *T*_0_ and a geographic region of receipt of care defined by the VA Integrated Network of Service. Missing values occurred in laboratory or vital sign measurements including 15.38% of the eGFR, 12.66% of the BMI and 11.70% of the blood pressure measurements. These missing values were imputed by conditional mean imputation based on the value within the group^41^. Continuous variables were transformed into restricted cubic spline functions to account for potential nonlinear relationships.

**Extended Data Fig. 9:**
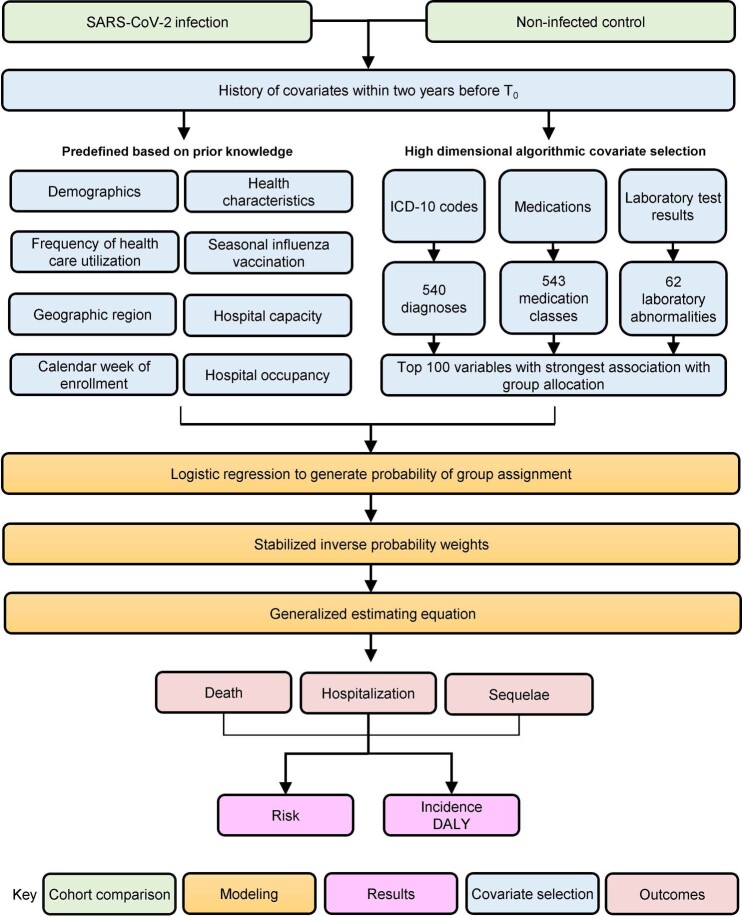
Analytic flowchart. Analytic flowchart of the study.

In addition to predefined covariates, we further included a set of algorithmically selected high-dimensional covariates as additional potential confounders to complement the list of prespecified covariates from data domains, including diagnoses, medications and laboratory results (Extended Data Fig. 9)^42^. Based on the Clinical Classifications Software Refined (CCSR), developed as part of the Healthcare Cost and Utilization Project sponsored by the Agency for Healthcare Research and Quality^43–45^, we classified more than 70,000 ICD-10 diagnosis codes into 540 diagnostic categories. We classified 3,425 medications into 543 medication classes on the basis of the VHA’s drug classification system^46,47^. On the basis of Logical Observation Identifiers Names and Codes, we classified 38 laboratory measures into 62 laboratory test abnormalities. From this list, we selected categories that occurred in at least 100 participants within each group, as rare conditions occurring in less than 100 participants (less than 0.5% in the smallest group with an *n* = 20,580) may not be sufficient to substantially describe the characteristics of the group. We then selected the top 100 variables that displayed the strongest univariate relative risk with group assignment and used them in addition to the predefined covariates in the models^48^.

### Statistical analyses

Baseline characteristics of the COVID-19 and control groups are presented as mean (s.d.), median (IQR) or frequency (%), as appropriate. An analytic flowchart is presented in Extended Data Fig. 9.

### Risks of death, hospitalization and prespecified sequelae

We first estimated risks by care setting of the acute phase of the disease, where analyses separated the COVID-19 cohort into two mutually exclusive groups based on if participants were nonhospitalized or hospitalized during the acute phase of the disease (first 30 days following infection of SARS-CoV-2). To estimate the risks of prespecified sequelae, we built subcohorts of those with no prior history of each sequela examined. We then estimated the probability of group membership using a series of logistic regressions where predictors included the predefined and high-dimensional covariates. The propensity scores were then used to calculate a stabilized inverse probability weight for use in weighting a subsequent analytic model. Covariate balance after the application of weights was assessed at baseline by the SMD, where a difference of less than 0.1 was taken as evidence of good balance^49^. We estimated the risks of sequelae in the weighted cohort during five different time periods of follow-up (30–90, 91–180, 181–360, 361–540 and 541–720 days after infection). To enhance consistency in the duration of follow-up between groups, we required that for a given period the participant had to have been followed through the full duration of that period (not censored due to death or end of follow-up). Relative risks were estimated from weighted generalized estimating equations using a log link and a binomial distribution, where the model included a time period and an interaction between the time period and the COVID-19 group. Incidence rates of a sequela due to COVID-19 were estimated by the difference in incidence rates in those with COVID-19 and the control group.

To more comprehensively capture the burden of each sequela and to facilitate a comparative assessment of the magnitude of health loss across all sequelae, we also estimated DALYs for each prespecified outcome on the basis of the product of its incident rate and its health burden coefficient (Supplementary Table 16)^23,24,50^. For each outcome, the estimate of the DALYs due to a sequela of COVID-19 was computed from the difference in the corresponding DALYs between the COVID-19 and control groups^23,24,51–53^.

CIs were developed by 1,000 times parametric bootstrapping based on covariates matrix generated from the generalized estimating equations. Incidence and DALYs are presented as rates per 1,000 persons. Risks are presented for comparisons of those with COVID-19 nonhospitalized and hospitalized during the acute phase of the disease and the control and in comparison of those with COVID-19 hospitalized versus nonhospitalized.

### Risks of sequelae overall and by organ system

We then examined the risks of several composite outcomes that aggregated sequelae into an overall outcome of PASC and sequelae by organ systems, including cardiovascular disorders, coagulation and hematologic disorders, diabetes, fatigue, gastrointestinal disorders, kidney disorders, mental health disorders, musculoskeletal disorders, neurologic disorders and pulmonary disorders. We estimated the incidence rates of having at least one sequela in that organ system. We also estimated the DALYs from sequelae in an organ system as the summation of the DALYs of sequelae within that organ system. This approach accounts for the number of sequelae experienced by an individual participant and the influence of each sequela (comparative to other diseases) on health^23,24,53^. Analyses of incidence at least one sequela within an organ system was conducted using generalized estimating equation with a log link and a logistic distribution. In analyses of risks and DALYs from sequelae within an organ system, we used generalized estimating equation with a log link and a Poisson distribution. Plots of smoothed cumulative DALYs were developed using penalized beta-splines.

### Risks in those with COVID-19 overall

Analyses were repeated in the overall COVID-19 cohort. Analyses were done for the individual sequelae, as well as for composite outcomes.

### Sensitivity analyses

We conducted several alternate versions of the analyses of risk of PASC in those with COVID-19 compared to the noninfected control to examine if a change in the analytic specification would change the results on risk of PASC (the primary outcome in this study). (1) As an alternative to the primary analytic approach of adjusting for confounding through inverse probability weighting, we used a conditional modeling-based approach that adjusted for baseline characteristics in each time period of analysis to account for changes in the composition of the cohort as follow-up progressed (*n* = 6,124,045) (ref. ^54^). (2) In addition to adjusting for the predefined and algorithmically selected high-dimensional baseline characteristics, in consideration of evidence that suggests that vaccination after SARS-CoV-2 infection may reduce the risk of adverse health outcomes^55^, we adjusted for vaccination status as a time-varying covariate, defined as 0, 1, 2 or 3+ more vaccination shots (*n* = 6,124,045). (3) As evidence has suggested that reinfection may add to risks of sequelae in addition to risks associated with the primary infection^42^, as an alternative approach to outcome assessment, we also censored participants at the time of reinfection (*n* = 6,124,045). (4) In addition to adjustment for baseline covariates, in consideration of potential differences in healthcare resource utilization between groups, we adjusted for the probability of having a healthcare encounter during follow-up (*n* = 6,124,045). The probability was estimated as the time-varying probability of having a healthcare encounter in each time period. (5) To assess the sensitivity of study results to differences in healthcare utilization over the course of study follow-up, we conducted the analysis in a subcohort of those that had at least one healthcare encounter during each time period of analysis in the course of follow-up (*n* = 4,961,017). (6) To examine the influence of iteration of bootstrapping, we conducted 500 iterations of bootstrapping instead of 1,000 iterations used in the primary approach (*n* = 6,124,045).

To further assess the sensitivity of the results to the effect of potential difference in censorship between the two exposure groups on risk estimates, we conducted analyses in those in the overall COVID-19 group compared to the noninfected control to estimate the relative risks while including all those who were censored during each time period (*n* = 6,124,045). We also used survival models to estimate the hazard ratios (*n* = 6,124,045).

### Negative outcome control

We examined the association between COVID-19 and incident neoplasms during the 2 years of follow-up as a negative outcome control. There is no prior knowledge supporting a causal association between COVID-19 and neoplasms. A successful application of negative control yielding the expected a priori null association between COVID-19 and risk of neoplasms may reduce (but does not eliminate) concerns about possible biases of the study^56^.

### Other analytic considerations

In all analyses, a 95% CI of risk that excluded unity as well as incidence and DALYs rates that excluded zero were considered evidence of statistical significance. CCSR version 2021.1 was used to classify diagnosis codes. Analyses were conducted using SAS Enterprise Guide version 8.2 (SAS Institute), and results were visualized using R version 4.0.4.

### Reporting summary

Further information on research design is available in the Nature Portfolio Reporting Summary linked to this article.

## Online content

Any methods, additional references, Nature Portfolio reporting summaries, source data, extended data, supplementary information, acknowledgements, peer review information; details of author contributions and competing interests; and statements of data and code availability are available at 10.1038/s41591-023-02521-2.

## Supplementary information

Reporting Summary

Supplementary Tables Supplementary Tables 1–16.

## Data Availability

The data that support the findings of this study are available from the US Department of Veterans Affairs. VA data are made freely available to researchers behind the VA firewall with an approved VA study protocol. For more information, please visit https://www.virec.research.va.gov or contact the VA Information Resource Center (VIReC) at VIReC@va.gov.

## Code availability

The analytic code is available at https://github.com/BcBowe3.
