# Supplementary material for: Postacute sequelae of COVID-19 at 2 years
Source: Nat Med. 2023 Aug 21;29(9):2347–57. doi: 10.1038/s41591-023-02521-2 (PMC10504070; doi:10.1038/s41591-023-02521-2)
Supplement: Supplementary file 1 — Reporting Summary [file 41591_2023_2521_MOESM1_ESM.pdf]

Corresponding author(s): Ziyad Al-Aly
Last updated by author(s): Jul 27, 2023

Reporting Summary

Nature Portfolio wishes to improve the reproducibility of the work that we publish. This form provides structure for consistency and transparency in reporting. For further information on Nature Portfolio policies, see our Editorial Policies and the Editorial Policy Checklist.

Statistics

For all statistical analyses, confirm that the following items are present in the figure legend, table legend, main text, or Methods section.

n/a	Confirmed	
☐	☒	The exact sample size (*n*) for each experimental group/condition, given as a discrete number and unit of measurement
☐	☒	A statement on whether measurements were taken from distinct samples or whether the same sample was measured repeatedly
☐	☒	The statistical test(s) used AND whether they are one- or two-sided<br>*Only common tests should be described solely by name; describe more complex techniques in the Methods section.*
☐	☒	A description of all covariates tested
☒	☐	A description of any assumptions or corrections, such as tests of normality and adjustment for multiple comparisons
☐	☒	A full description of the statistical parameters including central tendency (e.g. means) or other basic estimates (e.g. regression coefficient) AND variation (e.g. standard deviation) or associated estimates of uncertainty (e.g. confidence intervals)
☐	☒	For null hypothesis testing, the test statistic (e.g. *F*, *t*, *r*) with confidence intervals, effect sizes, degrees of freedom and *P* value noted<br>*Give P values as exact values whenever suitable.*
☒	☐	For Bayesian analysis, information on the choice of priors and Markov chain Monte Carlo settings
☒	☐	For hierarchical and complex designs, identification of the appropriate level for tests and full reporting of outcomes
☒	☐	Estimates of effect sizes (e.g. Cohen's *d*, Pearson's *r*), indicating how they were calculated

Our web collection on statistics for biologists contains articles on many of the points above.

Software and code

Policy information about availability of computer code

Data collection	SAS Enterprise Guide version 8.2 was used to collect data for the study.
Data analysis	Clinical Classifications Software Refined (CCSR) version 2021.1 were used to classify diagnosis codes. All analyses were done using SAS Enterprise Guide version 8.2 (SAS Institute, Cary, NC). Data visualizations were performed in R 4.0.4 (R Foundation for Statistical Computing, Vienna, Austria). Analytic codes are available at https://github.com/BcBowe3

For manuscripts utilizing custom algorithms or software that are central to the research but not yet described in published literature, software must be made available to editors and reviewers. We strongly encourage code deposition in a community repository (e.g. GitHub). See the Nature Portfolio guidelines for submitting code & software for further information.

Data

Policy information about availability of data

All manuscripts must include a data availability statement. This statement should provide the following information, where applicable:

- Accession codes, unique identifiers, or web links for publicly available datasets
- A description of any restrictions on data availability
- For clinical datasets or third party data, please ensure that the statement adheres to our policy

The data that support the findings of this study are available from the US Department of Veterans Affairs. VA data are made freely available to researchers behind

the VA firewall with an approved VA study protocol. For more information, please visit https://www.virec.research.va.gov or contact the VA Information Resource Center (VIReC) at VIReC@va.gov

Human research participants

Policy information about studies involving human research participants and Sex and Gender in Research.

Reporting on sex and gender	Information on sex was collected based on patient-self reported sex in the electronic health record. In the study cohort, 90% of the patients were male. Sex was balanced between groups during analyses.<br>Following the SAGER guidelines, we did not perform post-hoc analyses based on sex because the low incident event rate of the sequelae at 2 years of follow-up would be insufficient for us to enable meaningful conclusions about sex differences.
Population characteristics	The study included participants with mean age 63 years, 73% white race, 18% black race and 90% male.
Recruitment	Participants were identified based on criteria for cohort enrollment as specified in the methods section -- including people with SARS-CoV-2 infection between March 1, 2020 and December 31, 2020 and alive 30 days after infection and the non-infected control which included people with no record of infection between March 1, 2020 and December 31, 2020.
Ethics oversight	This study was approved by the institutional review board of the VA St. Louis Health Care System (protocol number 1606333).

Note that full information on the approval of the study protocol must also be provided in the manuscript.

Field-specific reporting

Please select the one below that is the best fit for your research. If you are not sure, read the appropriate sections before making your selection.

☒ Life sciences ☐ Behavioural & social sciences ☐ Ecological, evolutionary & environmental sciences

For a reference copy of the document with all sections, see nature.com/documents/nr-reporting-summary-flat.pdf

Life sciences study design

All studies must disclose on these points even when the disclosure is negative.

Sample size	To achieve better precision of the study results, we enrolled all users of the US Veterans Health Administration. 138,818 participants with COVID-19 and 5,985,227 non-infected control participants were enrolled
Data exclusions	To examine the risk of sequelae associated with SARS-CoV-2 infection, we predefined our exclusion criteria and excluded participants who did not survive the first 30 days of the SARS-CoV-2 infection.
Replication	The finding was not replicated because no external dataset with similar features is available to us.
Randomization	We conducted an observational study. Exposure allocation was not random. To balance the exposure groups, both predefined and algorithmically selected covariates were adjusted for through the inverse probability weighting methods.
Blinding	We conducted an observational study. Blinding was not possible.

Reporting for specific materials, systems and methods

We require information from authors about some types of materials, experimental systems and methods used in many studies. Here, indicate whether each material, system or method listed is relevant to your study. If you are not sure if a list item applies to your research, read the appropriate section before selecting a response.

Materials & experimental systems

n/a	Involved in the study	
☒	☐	Antibodies
☒	☐	Eukaryotic cell lines
☒	☐	Palaeontology and archaeology
☒	☐	Animals and other organisms
☒	☐	Clinical data
☒	☐	Dual use research of concern

Methods

n/a	Involved in the study	
☒	☐	ChIP-seq
☒	☐	Flow cytometry
☒	☐	MRI-based neuroimaging
